# The length and strength of compartmental interactions are modulated by condensin II activity

**DOI:** 10.1371/journal.pgen.1011724

**Published:** 2025-07-01

**Authors:** Randi Isenhart, Son C. Nguyen, Leah Rosin, Weihuan Cao, Patrick Walsh, Haris Muzaffar, Christopher E. Ellison, Eric F. Joyce

**Affiliations:** 1 Department of Genetics, Perelman School of Medicine, University of Pennsylvania, Philadelphia, Pennsylvania, United States of America; 2 Penn Epigenetics Institute, University of Pennsylvania, Philadelphia, Pennsylvania, United States of America; 3 Department of Genetics and Human Genetics, Institute of New Jersey, Rutgers University, Piscataway, New Jersey, United States of America; Geisel School of Medicine at Dartmouth, UNITED STATES OF AMERICA

## Abstract

The spatial organization of the genome is crucial for its function and integrity. Although the ring-like SMC complex condensin II has a well-documented role in organizing mitotic chromosomes, its function in interphase chromatin structure has remained more enigmatic. Using a combination of Oligopaint fluorescence in situ hybridization (FISH) and Hi-C, we show that altering condensin II levels in diploid *Drosophila* cells significantly changes chromosome architecture at large length scales between chromatin compartments. Notably, condensin II overexpression disrupts the robust boundary between heterochromatin and euchromatin, leading to interactions that span entire chromosomes. These interactions occur independent from transcriptional changes, suggesting that the mechanisms driving compartment formation and their interactions might be distinct aspects of genome organization. Our results provide new insights into the dynamic nature of chromosome organization and underscore the importance of condensin II in maintaining genomic stability.

## Author summary

The three-dimensional organization of the genome plays a critical role in regulating cellular function, yet the mechanisms that govern large-scale chromatin architecture during interphase remain incompletely understood. While the condensin II complex is well known for its function in chromosome condensation during mitosis, its role in shaping interphase chromatin has been less clear. In this study, we manipulated condensin II levels in diploid Drosophila cells and found that it directly modulates the strength and genomic span of chromatin compartment interactions. Elevated condensin II activity promoted extensive long-range intra-chromosomal interactions, including aberrant contacts between heterochromatin and euchromatin domains, whereas condensin II depletion preferentially disrupted distal interactions without altering compartment identity or gene expression. These results reveal that condensin II contributes to genome organization by facilitating long-range interactions independently of chromatin state or transcriptional output. Our findings support a model in which condensin II acts early in interphase to shape higher-order genome folding and maintain compartment boundaries, particularly near pericentromeric regions. This study expands our understanding of the molecular mechanisms underlying interphase genome architecture and highlights condensin II as a critical regulator of large-scale chromatin organization.

## Introduction

The genome contains different levels of organization that are essential in determining cellular functions. One crucial protein family in this process is the SMC (Structural Maintenance of Chromosomes) family, which includes cohesin and condensin (I and II) complexes [1,2]. Through their loop-extrusion activity, these complexes facilitate the higher-order organization of chromosomes [3,4]. Condensin I and II cooperate to facilitate this function during mitosis whereas cohesin is considered to be the primary loop extruding enzyme required during interphase to form chromatin loops and (topologically associating domains) TADs [5]. Importantly, cohesin loss has minimal impact on larger structures such as compartments and chromosome territories (CTs), leading to a popular model in which this scale of chromosome organization might occur independently of loop extrusion [6,7].

In contrast to cohesin, however, condensin is either absent or kept at relatively low levels during interphase by multiple mechanisms [8,9]. Despite this, emerging evidence points to a significant role for condensin II in interphase chromosome organization, including regulation of chromosome size and local genomic interactions between neighboring A compartments [10–15]. Increasing condensin II levels during interphase also exhibits dramatic effects [8], leading to smaller CTs that intermix less, reducing homolog pairing and translocation rates in the process [10,12–14,16–20]. These results suggest that condensin II can impact genomic interactions at multiple length scales during interphase and may be tightly regulated. However, the exact nature of condensin II interactions and how they relate to specific chromatin types remains unclear.

Here, we manipulated the levels of condensin II during interphase in diploid Drosophila BG3 cells. We then examined the resulting changes in intra-chromosomal interactions using a combination of chromatin state-specific painting and high-resolution Hi-C analysis. Our findings suggest that condensin II-mediated long-range interactions are critical for higher-order contacts between compartments without altering compartment designation, which is in stark contrast to cohesin depletion that enhance these interactions. Furthermore, using a custom genome-build from long-read sequencing to map pericentric regions following Hi-C, we find that when condensin II is overexpressed during interphase, prominent stripes of interactions emanate from pericentric domains and spread across entire chromosomes. In summary, this study sheds new light on the role of condensin II in the organization of the genome at large length scales, emphasizing the importance of long-range looping interactions in proper chromosome folding.

## Results

### Condensin II shapes chromosomes in a chromatin type-nonspecific manner

**Fig 1:**
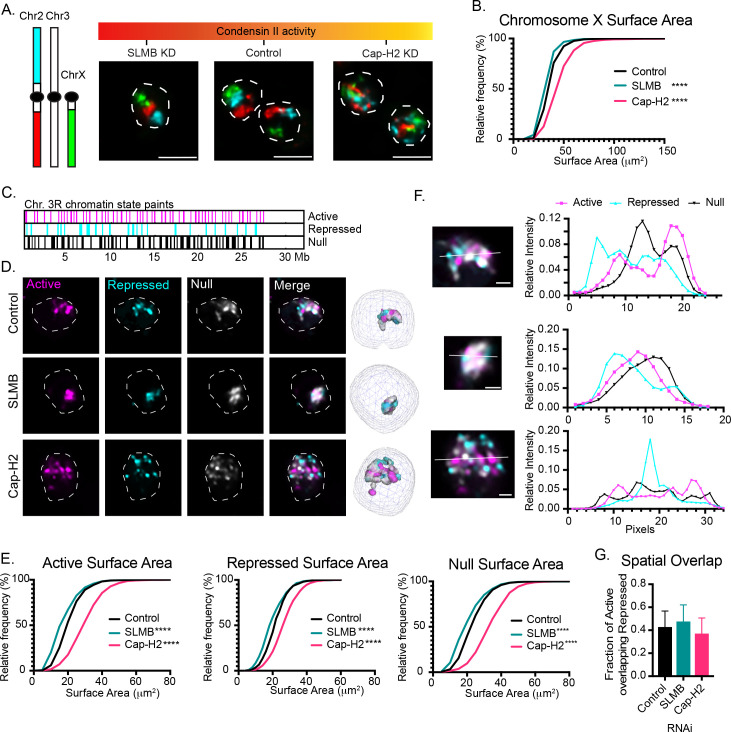
Condensin II shapes chromosomes in a chromatin type-nonspecific manner. A. Oligopaints FISH probes label the unique sequences of chromosomes 2L, 2R, and X in red, cyan, and green, respectively. Example nuclei from SLMB KD, control (brown KD), and Cap-H2 KD with gradient schematic indicating relative amounts of condensin II in each condition. Dotted lines represent nuclear boundaries defined by Hoescht staining. Scale bar represents 5 µm. B. Cumulative frequency histograms of chromosome X surface for control (brown KD), Cap-H2 KD, and SLMB KD conditions. N: Control = 1229, Cap-H2 = 1050, SLMB = 879. **** represents p < 0.0001 in Mann-Whitney test. C. Chromatin state probes label chromosome 3R based on underlying chromatin state. Note: resolution scaling doesn’t show all regions. See S1 Table for full map of probed regions. D. Example nuclei from control (brown KD, top row), SLMB KD (second row), and Cap-H2 KD (third row) labeled with chromatin state probes. The first three columns show signal from individual channels and the fourth column merges all three channels. Dotted lines represent nuclear boundaries defined by Hoescht staining. Scale bar represents 5 µm. The final column represents the 3D segmentation of each channel. E. Cumulative frequency histograms representing the surface area of each segmented structure per nucleus. N: Control = 1419, Cap-H2 = 1331, SLMB = 743. **** represents p < 0.0001 in Mann-Whitney test. F. Zoom-in of the chromosomes from Fig 1D, column 4. Scale bar represents 1 µm. Signal intensity was calculated along the drawn line. The relative intensity from the various channels were calculated for each pixel along the line and plotted in the second column. G. The fraction of repressed (polycomb) volume overlapping with the active domain was calculated for each nucleus. Bar represents mean and error bars represent one standard deviation. N: Control = 1419, Cap-H2 = 1331, SLMB = 743.

In both Drosophila and mammalian systems, condensin II activity is actively inhibited during interphase [8,9]. To increase condensin II activity in Drosophila, we treated diploid BG3 cells with siRNA targeting SLMB, an F-box protein that targets the condensin II subunit Cap-H2 for degradation during interphase [8,13]. This led to an 83% decrease in SLMB transcripts and 183% increase in Cap-H2 protein levels (S1 Fig). For comparison, we directly targeted Cap-H2 by siRNA, which resulted in a 50% reduction in transcripts and 75% reduction in protein levels (S1 Fig). We then utilized Oligopaint FISH probes to simultaneously label the unique portions of chromosomes 2L, 2R, and X to measure the volume of each chromosome territory (CT) across each condition (Fig 1A). Consistent with our previous work in tetraploid Kc167 cells [14], SLMB and Cap-H2 knockdown (KD) resulted in either decreased or increased surface areas of each chromosome, respectively (Figs 1B and S1), indicating that altered Cap-H2 levels during interphase were sufficient to change large-scale chromosome folding patterns across multiple Drosophila cell types.

We reasoned that condensin II may influence CT size in a chromatin-type-specific or -nonspecific fashion. Previous ChIP-seq data revealed that Cap-H2 is enriched at architectural protein binding sites in elongating gene bodies and active compartments, suggesting Cap-H2 may specifically affect active regions [10,21]. However, in addition to active chromatin (44% of peaks), Cap-H2 peaks can be found within designated repressed (15% of peaks) and null (41% of peaks) chromatin types as defined by epigenetic marks (S1 Fig) [10,22].

To determine if a specific chromatin type accounts for the majority of chromosome expansion following Cap-H2 KD, we developed a novel Oligopaint FISH assay to label the entire unique portion of a chromosome in three colors based on the underlying chromatin state. Specifically, as previously determined through unsupervised clustering of 403 epigenetic profiles [22], we labeled active, repressed (polycomb), and null (remaining) chromatin across chromosome 3R and segmented each structure in 3D (Fig 1C and 1D, and S1 Table). Similar to our analysis of whole chromosomes, SLMB and Cap-H2 KD resulted in a significant decrease or increase in surface area for all three chromatin types, respectively (Figs 1E and S2). The number of segmented structures per nucleus was also altered, with increased and decreased numbers following SLMB and Cap-H2 KD, respectively (S2 Fig). Together, this indicates that condensin II is both necessary and sufficient to alter the folding of CTs in a chromatin-type-independent and therefore gene expression-independent manner.

Interestingly, when signals from each chromatin type were overlayed with one another, we observed signal peaks at different positions across each CT, consistent with spatial separation of the three chromatin types in individual nuclei [23,24] (Fig 1F). However, despite changes in signal size, we observed only minor changes in intermixing between active and repressed chromatin in SLMB or Cap-H2 KD cells (Figs 1G and S2), suggesting condensin II influences the compaction of different chromatin types without dramatically altering their spatial separation.

### Condensin II preferentially influences long-range intra-chromosomal interactions

To better understand condensin II’s role in chromosome compaction, we performed Hi-C in *Drosophila* BG3 cells following knockdown of Cap-H2 or SLMB in two biological replicates each. Unlike previous studies conducted in polyploid cells [21], BG3 cells are stably diploid, allowing us to more accurately detect long-range interactions without the confounding effects of polyploidy that may impact these longer-range interactions [25].

**Fig 2:**
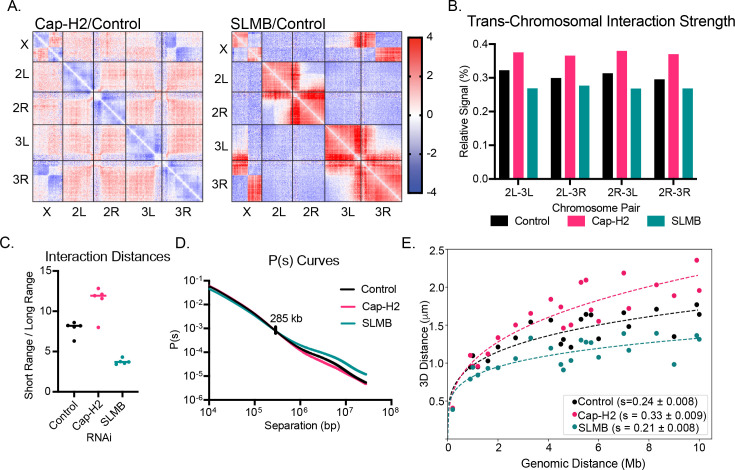
Condensin II preferentially influences long-range intra-chromosomal interactions. A. Hi-C map reflecting Cap-H2 KD normalized to control (brown KD) (first panel) and SLMB KD normalized to control (second panel). B. Percent of total reads/condition reflecting interactions between each chromosome pair. C. Ratio of short-range (<1Mb) to long-range (≥1Mb) contacts per chromosome. Each data point represents one chromosome arm. D. Probability of contact curve for each Hi-C dataset. Control and SLMB lines intersect at 285kb. E. Map of the 22 probed pairs of 100kb regions along chromosome 3R. F. 3D-distance from 22 probed pairs of regions plotted as function of genomic distance and fitted with power-law curves.

Across all samples, we noted an X-3R translocation in a subpopulation of cells, as previously described in BG3 cells [25]. We generated interaction maps for each condition, normalized to a control (Fig 2A). In the Cap-H2 KD, differential contact maps show a broad increase in inter-chromosomal (both trans-chromosomal and inter-arm) interactions and a decrease in intra-chromosomal interactions (Figs 2A and 2B, and S3), consistent with chromosome decompaction as shown above and by previous FISH or Hi-C analyses [12,14,20,26]. In contrast, the SLMB KD differential contact map displayed a near-mirror image of Cap-H2 KD with a significant decrease in trans-chromosomal interactions and an increase in intra-chromosomal interactions (Figs 2A and 2B, and S3).

For each chromosome arm, we quantified the shift in interaction strength by calculating the ratio of short- (<1Mb) to long-range (≥1Mb) contacts (Fig 2C). The Cap-H2 KD shifted the ratio towards shorter-range contacts, while the SLMB KD shifted the ratio towards longer-range contacts for each chromosome arm. The probability of contact at different length scales along the chromosome arm, as shown by the P(s) curves, further revealed that regions beyond 285kb are most affected by condensin II levels, with the maximum effect size beyond 1Mb (Fig 2D). Importantly, we noted that shorter-range interactions below this inflection point displayed the opposite phenotypes: enhanced following Cap-H2 KD and reduced following SLMB KD. These short-range results are consistent with previous findings that suggested Cap-H2 KD increases intra-genic and short-range compartmental interactions [10].

Focusing on the long-range interactions, we sought to test whether this length-dependent sensitivity could be detected within individual cells. We performed FISH targeting 22 pairs of loci at varying distances along chromosome 3R (S4 Fig). Following Cap-H2 KD, we observed decreased contact frequency and increased 3D-distance between regions >1Mb apart (S4 Fig). Conversely, following SLMB KD, we noted an increased contact frequency and decreased 3D-distance between regions >1Mb apart (Figs 2E and S4). No significant changes were detected for either KD when loci were 200Kb apart (S4 Fig). Fitting a power-law curve to the 3D distance as a function of the genomic distance showed that the scaling factor of chromosomes was altered following either KD, indicating that the magnitude of contact changes for each KD was dependent on the genomic distance between the probed regions (Fig 2E). These results suggest that, at the highest orders of organization, chromosomes are unfolding when Cap-H2 levels are decreased and exhibit enhanced folding when Cap-H2 levels are increased during interphase.

### The strength and distance of compartment interactions scale with condensin II levels

**Fig 3:**
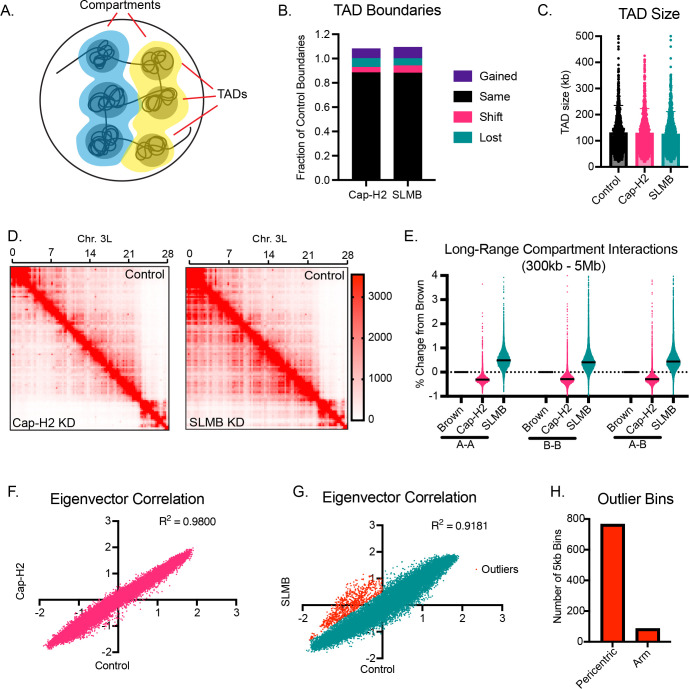
The strength and distance of compartment interactions scale with condensin II levels. A. Cartoon schematic of TADs and compartments within a nucleus. B. Quantification of TAD boundary changes across Hi-C datasets at 5kb. “Shift” refers to boundaries that have shifted 15-70kb. “Same” includes boundaries that shifted ≤10kb. “Lost” boundaries are present only in the control condition. “Gained” boundaries are present only in the KD condition. 918 total TADs were detected in brown KD, 924 TADs in Cap-H2 KD, and 945 TADs in SLMB KD. C. Quantification of TAD size. Each data point represents one TAD, bar represents mean, and error bar represents standard deviation. Conditions are not statistically significantly different from control in Mann-Whitney test. D. Hi-C map of chromosome 3L at 50kb resolution, balanced. Above the diagonal reflects signal in the control (brown KD) condition; below the diagonal reflects signal in the Cap-H2 KD (left panel) or SLMB KD (right panel). E. Quantification of pairwise compartmental interactions, graphed as percent change from control. Only compartment pairs that were between 500kb and 5Mb were included. F. Correlation of the PC1 values of the compartment eigenvector analysis at 5kb resolution from control and Cap-H2 KD. R^2^ = 0.9800. G. Correlation of the PC1 values of the compartment eigenvector analysis at 5kb resolution from control and SLMB KD. R^2^ = 0.9181. Red represents outlier data points that were B comparements in control and where the difference between control and SLMB was at least -0.6. H. Location of the outlier B-to-A or B-to-weaker B bins in Fig 3G (red data points) as either in the pericentric domain or the chromosome arm.

The distance-dependent nature of the condensin II phenotype led us to examine whether there were preferential changes in either of the two distinct features of higher-order chromosome folding: TADs (topologically associating domain) or compartments (Fig 3A). We defined TAD boundaries at 5kb resolution across conditions and found that 88.3% and 83.1% of boundaries did not change by more than 10kb following Cap-H2 or SLMB depletion, respectively (Fig 3B). The size of TADs also did not vary across conditions (Fig 3C; control average = 130kb, N = 918; Cap-H2 average = 129kb, N = 924; SLMB average = 125kb, N = 945). This suggests that condensin II levels have little effect on TADs, consistent with the average TAD size (130kb) being below the scale in which we observed global changes in interactions (285Kb).

Next, we analyzed chromatin compartments and generated eigenvectors to assign sequences to either the A or B compartment. We focused on non-adjacent compartments and calculated an inter-compartment contact score for each pair of compartments located at least 300kb apart. The interaction strength for each condition was normalized to its interaction score in controls. As evident in the Hi-C matrices (Fig 3D), we found distance-dependent decreased and increased interactions between compartments in the Cap-H2 and SLMB KDs, respectively, regardless of whether they were designated as A-A or B-B compartment interactions (Fig 3D and 3E). Similar results were found when we segmented the Hi-C data based on the underlying chromatin state (active, repressed, and null) (S3 Fig). Furthermore, while less frequent contacts are observed between A-B compartment pairs in general, the differences were similar to those between A-A or B-B pairs (Fig 3E), supporting our imaging data in showing that condensin II influences interactions between long-range compartment domains in a chromatin-type-independent manner.

Despite changes in the length and strength of compartmental interactions, we observed a high correlation of compartment designation between the Cap-H2 KD and control cells, with an R^2^ value of 0.9800 (Fig 3F). Consistent with this conclusion, we observed minimal changes in gene expression via RNA-seq after Cap-H2 KD (S5 Fig). Specifically, Cap-H2 KD resulted in the downregulation of only 4 genes and the upregulation of 24 genes and 3 transposable elements out of 8,806 detected features. Importantly, this suggests that the large-scale organizational changes following Cap-H2 KD are occurring independently of changes in gene expression. By comparison, in the SLMB KD, we found a slightly reduced correlation of compartment designation with an R^2^ value of 0.9181 (Fig 3G), owing to a subset of 5kb bins that significantly changed as compared to control cells. These bins (Fig 3G, red) were all classified as B compartments in control cells and called as a weaker B or A following SLMB depletion. Interestingly, ~ 90% of these bins were located within the pericentric heterochromatic ends of each chromosome arm (Fig 3H). However, similar to Cap-H2 depletion, minimal changes in gene expression were found for genes located within these regions (S5 Fig).

Together, these data indicate that the majority of the genome does not undergo changes in gene expression or compartment designation following either acute gain or loss of condensin II activity. Instead, levels of condensin II equally influence the distal interactions between A-A and B-B compartment pairs with a preferential sensitivity near the pericentric ends of each chromosome.

### Condensin II overexpression disrupts the boundary between the pericentromeric heterochromatin and euchromatic chromosome arm

**Fig 4:**
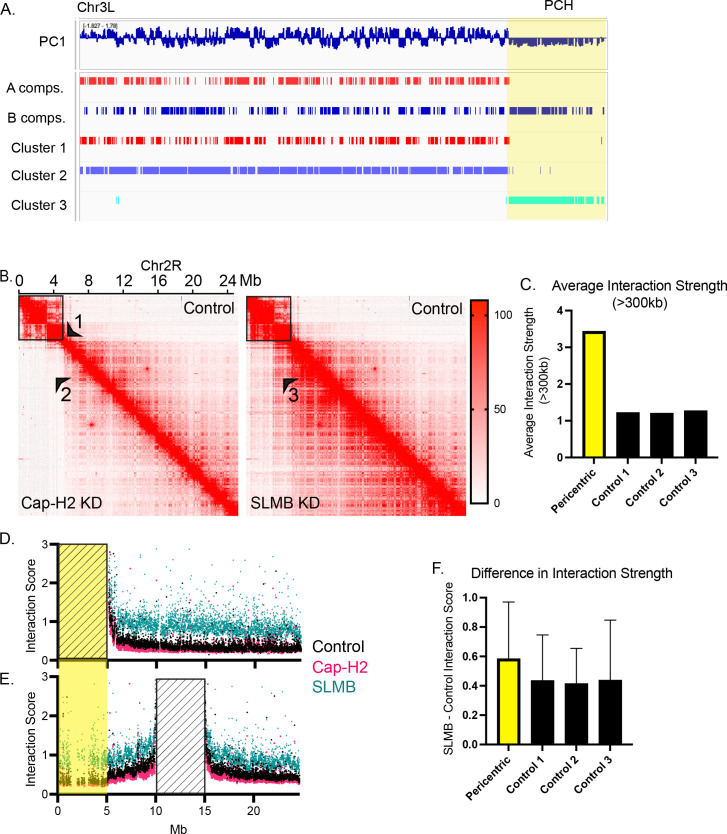
Condensin II overexpression disrupts the boundary between the pericentromeric heterochromatin and euchromatic chromosome arm. A. Map of chromosome 3L in entirety. The first track indicates the first principal component of the Homer eigenvector analysis, with positive values representing A-like state and negative values representing B-like state. The second and third tracks indicate A and B compartments as designated by Homer. The final three tracks indicate k-means clusters called from the first three eigenvectors from cooltools. The highlighted region indicates the defined pericentric heterochromatin domain. B. Hi-C map of chromosome 2R at 10kb resolution, balanced. Above the diagonal reflects signal in the control condition, below the diagonal reflects signal in the Cap-H2 KD. Box represents the 5Mb pericentric compartment on chromosome 2R. Arrowhead 1 indicates reduced interactions between the pericentric compartment and the rest of the arm in control. Arrowhead 2 indicates further reduced interactions between the pericentric compartment and the rest of the arm in the Cap-H2 KD. Arrowhead 3 indicates enhanced interactions between the pericentric compartment and the rest of the arm in the SLMB KD. C. Average strength of interactions above 300kb in chromosome 2R pericentric compartment (yellow) and sized-matched control regions along the arm (black). D. Virtual 4-C using the 5Mb pericentric compartment as an anchor. Yellow highlighted region represents the pericentric compartment and diagonal black lines represent the anchor. Each data point represents the strength of interaction between the anchor and a 5kb bin along the length of chromosome 2R. E. Virtual 4-C using a control 5Mb region as an anchor. Yellow highlighted region represents the pericentric compartment and diagonal black lines represent the anchor. Each data point represents the strength of interaction between the anchor and a 5kb bin along the length of chromosome 2R. F. Quantification of the virtual-4C data. The difference between SLMB and control was calculated for each 5kb in Fig 4D and the mean and standard deviation of that difference are represented by the yellow bar. The difference between SLMB and control for each 5kb bin in Fig 4E are represented by the second bar (Control 1). The same was done for two additional control regions (Control 2 and 3).

To further explore the interactions within and emanating from the pericentric heterochromatin, we generated a BG3-specific genome build via nanopore sequencing to accurately map our Hi-C data to this portion of the genome (see Methods). The BG3-specific build was 99% identical to the dm6 genome build at the nucleotide level but contained >5,000 differences in repeat insertions, amounting to over 9Mb of genomic content. Because of the high similarity between builds, we noted no differences in the Hi-C results presented thus far. We were, however, able to resolve 1–5Mb of pericentromeric chromatin on four out of the five main chromosome arms, including chromosome 3L, as shown in Fig 4A (yellow region). We were unable to resolve a pericentromeric region on chromosome X, likely due to the X-3R translocation present in a subpopulation of the BG3 cell line. We next used K-means clustering on the first three principal components of the eigenvector analysis to define three compartment types instead of two (Fig 4A). This independently revealed the pericentric compartments (P compartments), as clustering separately from the A and B compartments along each chromosome arm (S2 Table). As previously shown in other studies [27–29], P compartments displayed reduced interactions with the rest of the chromosome arm, owing to a singular boundary near the pericentric ends of each chromosome (Fig 4B, arrowhead 1). In contrast, interactions within the P compartment are enhanced compared to size-matched randomly selected control regions more distal along the arm (Fig 4C), indicating that the P compartments are strongly self-interacting domains.

Albeit subtle, Cap-H2 KD further enhances the separation of the P compartment from the rest of the chromosome arm, consistent with its KD reducing long-range interactions in general (Fig 4B, arrowhead 2). However, SLMB KD dramatically increased interactions between the P compartment and the rest of the chromosome arm (Fig 4B, arrowhead 3). We quantified these changes using a virtual-4C approach anchored at the entire 5Mb P compartment on chr2R (Fig 4D). This shows that although SLMB KD enhances all interactions across the chromosome arm, it more dramatically increases interactions emanating from the P compartment as compared to randomly selected size-matched control regions (Fig 4D and 4E).

Interestingly, Cap-H2 and SLMB KD also exhibit their effects on interactions within the P compartment (S6 Fig). Following SLMB KD, P compartments become divided by small A compartments (S6 Fig). As mentioned above, these designation switches account for the majority of compartment switches in the genome following SLMB depletion, due to increased interactions between the P compartment and the euchromatic chromosome arm. These results are consistent with condensin II’s effects on compartment interactions in general and further highlight how its over-activity can break strong boundaries near the centromere that separate heterochromatic and euchromatic regions.

### Condensin II overexpression creates a pericentric stripe of interactions across the centromere

**Fig 5:**
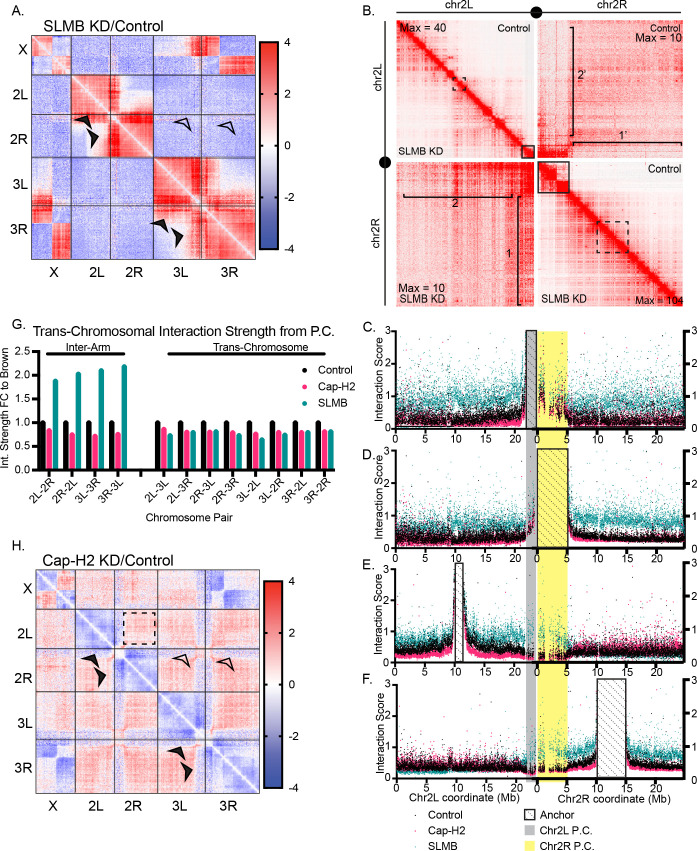
Condensin II overexpression creates a pericentric stripe of interactions in trans. A. Hi-C map reflecting SLMB KD normalized to control. Solid arrowheads indicate increased interactions from the pericentric compartment across the centromere. Hollow arrowheads indicate no increased interactions from the pericentric compartment to other chromosome arms. B. Hi-C map of chromosomes 2L and 2R with control shown above the diagonal and SLMB KD shown below the diagonal. Note that each quadrant is scaled separately for visualization purposes. The maximum value of each quadrant is listed in the outer corners. Solid boxes represent the pericentric compartments (1Mb on chromosome 2L and 5Mb on chromosome 2R). Dashed lines represent the size-matched randomly selected control regions along the chromosome arm. Brackets 1 and 1’ indicate trans interactions from the chr. 2L pericentric compartment in SLMB KD and control, respectively. Brackets 2 and 2’ represent trans interactions from the chr. 2L pericentric compartment in SLMB KD and control, respectively. C-F. Virtual-4C. Gray highlight indicates the chromosome 2L pericentric compartment, diagonal dashed lines represent the anchor, and yellow highlight represents the chromosome 2R pericentric compartment. Each data point represents the interaction strength between the anchor and a 5kb bin. Black represents control, pink represents Cap-H2 KD, and blue represents SLMB KD. C. Virtual-4C from chromosome 2R pericentric compartment. D. Virtual-4C from chromosome 2L pericentric compartment. E. Virtual-4C from chromosome 2R control region. F. Virtual-4C from the chromosome 2L control region. G. Interaction strength calculated from the pericentric compartments to other chromosomes, graphed as the fold change from control. Measurements reflect the pericentric compartment of the first listed chromosome on the x-axis to the entirety of the second listed chromosome. H. Hi-C map reflecting Cap-H2 KD normalized to Brown KD. Solid arrowheads indicate decreased interactions from the pericentric compartment across the centromere. Hollow arrowheads indicate decreased interactions from the pericentric compartment to other chromosomes.

We next examined inter-chromosomal interactions to determine how condensin II affects interactions between the P compartments and other chromosome arms. We noted dramatic stripe-like interactions on the SLMB KD contact maps emanating from P compartments that appear to interact with the entirety of the chromosome, including the inter-arm interactions across the centromere (Fig 5A, filled arrowheads and Fig 5B brackets 1 and 2 compared to brackets 1’ and 2’). To quantify these “pericentric stripes,” we expanded our virtual-4C analysis, focusing on interactions across both arms of the chromosome (Fig 5B, solid boxes to bracketed regions). For both the 1Mb chr2L P compartment (Fig 5C) and the 5Mb chr2R P compartment (Fig 5D), we observed increased contact along the entire length of both chromosome arms in the SLMB KD condition. In contrast, randomly selected size-matched control regions (Fig 5B, dashed boxes) only showed increased inter-arm interactions to the P compartment of the opposing arm (Fig 5E and 5F). Therefore, SLMB KD specifically enhances the inter-arm contacts from the P compartment despite reducing global inter-arm interactions.

Importantly, we found no evidence for increased trans-chromosomal contacts between chromosome 2 P compartments and any other chromosomes. Instead, trans-chromosomal interactions were broadly depleted following SLMB KD (Fig 5A and 5G hollow arrowheads), consistent with its role in promoting somatic homolog pairing [8,14,19,21]. These results suggest that SLMB KD enhances interactions from the P compartment to all regions along the same chromosome.

Conversely, Cap-H2 KD decreases all interactions emanating from the P compartment (Fig 5G and 5H, all arrowheads) despite the global increase in inter-chromosomal and trans-chromosomal interactions, consistent with its known role in antagonizing somatic homolog pairing [8,14,16,19,21] (Figs 5H (dashed box) and 2B). Within a chromosome, interactions from the chromosome 2 P compartments are reduced along the entire length (Fig 5H, solid arrowheads) as well as to the other chromosomes (Fig 5H, hollow arrowheads). These results suggest that Cap-H2 KD enhances the spatial separation between the P compartments and the chromosome arms despite globally increasing interactions between chromosome arms.

## Discussion

In this study, we characterize the pivotal role of condensin II in orchestrating long-range compartmental connections within the genome. Our findings show a direct relationship between the levels of condensin II and the extent and potency of these interactions. Remarkably, this relationship persists independently of chromatin structure and gene expression. Our study also demonstrates that the classification of chromosomal segments into A and B compartments remains remarkably stable in the absence of condensin II. Outside of the pericentric heterochromatin regions, condensin II loss does not lead to increased intermixing between these compartments. This observation suggests that the mechanisms driving compartment formation and their interactions might be distinct aspects of genomic organization, each subject to separate regulatory control.

By generating a genome build specific to our cell type, we discover condensin II’s pronounced influence on the pericentric heterochromatin region, which we refer to as the P compartment due to its previously shown reduced interactions with the rest of the chromosome arm [27–29]. Reduced levels of condensin II further enhance this separation between the P compartment and chromosome arms, despite an overall increase in inter-chromosomal interactions. In contrast, increased condensin II levels enhance contacts between the P compartment and both arms of each chromosome. Importantly, these effects occur at a larger scale than the condensin-dependent interactions between adjacent A-A compartments as previously described [10]. This scale of reorganization is also distinct from relatively short-range chromatin loops and topologically associating domains (TADs) typically steered by cohesin [6,15]. Indeed, we define an inflection point of ~300Kb wherein condensin II loss and gain switch phenotype directionality.

Our findings that Cap-H2 knockdown preferentially disrupts long-range interactions while increasing short-range contacts suggest a key role for condensin II in balancing chromatin organization at multiple scales. Although condensin II is widely recognized for its role in preventing homolog pairing in *Drosophila* [8,13,19], this function alone does not entirely account for its influence on long-range chromatin interactions. We observed similar effects on the single X chromosome in the male BG3 cells. Although analyzing these effects was complicated by the presence of an X-3R translocation, this observation suggests that condensin II plays a role in shaping large-scale chromatin architecture beyond just inhibiting homolog pairing. Furthermore, recent work [21] has shown that zinc-finger protein Z4 works in conjunction with Cap-H2 at architectural protein binding sites to regulate both somatic pairing and chromatin structure. Given that chromosome pairing is associated with stronger short-range intrachromosomal interactions, such as gene loops and A-compartment contacts [16,21], the increased short-range interactions observed after the depletion of Cap-H2 may indicate an overall loosening of chromatin architecture rather than being solely due to pairing effects.

**Fig 6:**
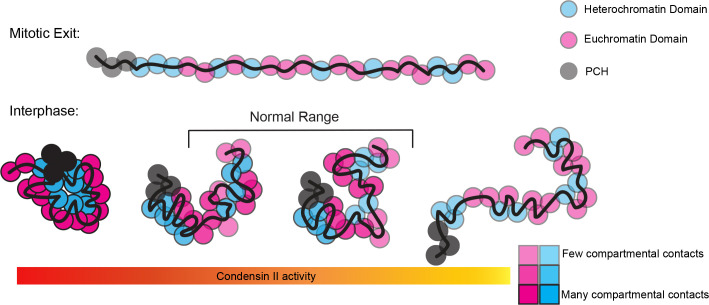
Condensin II activity directs interphase chromosome conformation. A. Condensin II is required to re-shape chromosomes from the Rabl configuration at mitotic exit into chromosome territories in early interphase. The degree to which this conformational change occurs depends on the level of condensin II activity. Increased condensin II activity (left) promotes more distal interactions permitting for biophysical properties to bridge more distal regions. Alternatively, reduced condensin II activity (right) fails to provide the prerequisite distal interactions, resulting in only local compartmental interactions. Thus, we propose that interphase chromosome conformation exists on a spectrum from Rabl-like interphase chromosomes to highly compacted chromosome territories, rather than discrete organizational types [31]. This spectrum of conformations is largely dependent on condensin II activity, which is heavily regulated during interphase.

Additionally, the disruption of long-range interactions implies that condensin II actively facilitates these contacts (Fig 6), possibly through mechanisms like loop extrusion. That an interphase-specific regulator of condensin II (SLMB) can impact these interactions suggests that condensin II has the capacity to extrude loops at this cell cycle stage. We propose a model in which a relatively low level of condensin II remains associated with chromatin at mitotic exit and loop extrudes over long distances, while chromosomes are mostly devoid of transcription and other complexes that may inhibit its extrusion. At this critical stage early in interphase, condensin II levels dictate how tightly or loosely the chromosome polymer will be folded throughout the remainder of the cell cycle (Fig 6). As interphase progresses, compartments coalesce, and other factors, including cohesin and transcriptional machinery may begin to fine-tune interactions at the TAD and loop level. These structures can form regardless of condensin II as they rely on chromatin regions in relatively close linear, and therefore 3D, proximity. Importantly, our model is consistent with condensin II being dispensable for maintaining long-range interactions once established, explaining why the consequences of condensin II loss require an intervening cell cycle to fully manifest [30,31].

While the function of condensin II-mediated interactions during interphase remains unclear, we speculate that they may create a favorable local environment for similar chromatin states to aggregate via an autonomous mechanism. While the prevalent models attribute compartmental interactions to protein aggregation influenced by biophysical characteristics [32–36], this model fails to explain how distal chromatin regions initially come into proximity and how compartmental interactions in general avoid excessive intermixing between chromosomes. Indeed, the enhanced homolog pairing and CT intermixing following Cap-H2 KD, as observed by many groups [10,13,16–19], suggests that condensin II acts to separate individual chromosomes, possibly to avoid these very interactions. A similar model has also been proposed for cohesin at much smaller genomic contexts [37]. In conclusion, our study elucidates the role of condensin II in shaping long-range compartmental dynamics within the genome and presents a novel perspective on the relationship between loop extrusion and compartmental interactions.

## Materials and methods

### dsRNA production

The primer sequences as noted in S4 Table were used to both amplify the gene of interest from genomic DNA and add T7 adapters. The resulting PCR products were purified using a NucleoSpin Gel and PCR Cleanup kit (Macherey-Nagel). dsRNA was generated using the MEGAscript T7 kit (Invitrogen) and purified using the RNeasy kit (Qiagen). dsRNAs were heated to 65°C for 30 minutes and then cooled slowly to room temperature to renature dsRNA.

### Cell culture and knockdowns

BG3 cells were obtained from the *Drosophila* Genome Resource Center (DGRC 66). Cells were grown at 25°C in M3 media, supplemented with 10% FBS and 10 µg/ml insulin. Cultures were split twice per week at a 1:4 ratio.

For knockdowns, 4x10^6^ cells were incubated with 20µg of dsRNA in 1mL of serum-free medium for 30 mins in each well of a six-well plate. After incubation, 3mL of complete medium was added to the cells. Cells were cultured for four days. Control cells were treated with dsRNA targeting *brown*.

### qPCR

RNA was extracted from cells using the RNeasy Kit (Qiagen) and converted to cDNA using the Maxima Reverse Transcriptase kit (Thermo Scientific). qPCR was run using PowerUp SYBR Green Master Mix (Applied Biosystems). Genes of interest were compared to the geometric mean of three housekeeping genes (Aldh7A1, P5CS, and Ssadh) using the Pfaffl method. Primers used are listed in S5 Table.

### Cap-H2-EGFP transfection with knockdowns

On day one, BG3 cells were seeded into 6-well plates and allowed to adhere overnight. On day two, cells were transfected with a plasmid containing Cap-H2-EGFP driven by an inducible metallothionien promoter using Lipofectamine 3000 (Invitrogen). The plasmid was a gift from Giovanni Bosco [8]. On day three, cells were treated with 20µg of dsRNA targeting brown, Cap-H2, or SLMB, as listed above. On day six, expression of Cap-H2-EGFP was induced with CuSO_4_ (1mM final concentration). On day 7, cells were harvested in RIPA buffer for western blots (100 µl per 2 million cells) or affixed to slides for FISH or IF.

### Western blotting

Whole cell lysate was mixed with NuPAGE LDS sample buffer and sample reducing reagent (Thermo Fisher Scientific), denatured at 70°C for 10 min, and cooled on ice. 20µg of sample was run on Mini-PROTEAN TGX Stain-free precast gels. The samples were transferred to a 0.2µm nitrocellulose membrane for 1 hour at 4°C then washed in TBST buffer (TBS buffer + 0.1% Tween-20). The membrane was blocked in 5% milk in TBST for 30 minutes at room temperature and then incubated with primary antibodies diluted in 5% milk TBST overnight at 4°C. GFP polyclonal antibody (Invitrogen A6455) was diluted 1:500 and alpha-tubulin monoclonal antibody (Invitrogen T6074) was diluted 1:1000. The next day, membranes were washed three times in TBST for 10 minutes each and then incubated with secondary antibodies diluted in 5% milk TBST at room temperature for 1 hour. HRP anti-rabbit IgG (Cell Signaling 7074) was diluted 1:10000 and HRP anti-mouse IgG (Cell Signaling 7076) was diluted 1:10000. The membranes were washed three times in TBST and once in TBS for 10 minutes each. Blots were then incubated for 4 minutes with Clarity Max ECL Western Blotting Substrate (Bio-Rad) and imaged on the ChemiDoc MP Imaging System and analyzed using Bio-Rad Image Lab software (v.5.2.1).

### Probe design and synthesis

Oligopaint libraries were designed as previously described, using the Oligoarray 2.1 software or OligoMiner [38,39] and the Dm3 genome build, and purchased from CustomArray/Twist Bioscience. Chromosome territory paints were from Rosin et. al 2018 [14] and were designed to have 42 bases of homology and a density of approximately 1probe/kb. Chromatin state probes were designed to label 27.59 Mb of unique sequence on chromosome 3R using the active, polycomb, and null chromatin state designations from Sexton, et. al 2012 [22] and were designed to have 42 bases of homology and a density of approximately 0.8 probe/kb. Coordinates for state probes can be found in S1 Table. Individual 100kb spots along chromosome 3R were designed to have 80 bases of homology and were directly dye-conjugated as described in Park, et. al. 2023 [40]. Coordinates for 100kb spot probes can be found in S3 Table. Chromosome territory and chromatin state probes were synthesized as described in Rosin et. al 2018 [14] and 100kb spot probes were synthesized as described in Park et. al 2023 [40].

### FISH

Cells were settled onto poly-L lysine coated glass slides at a concentration of 1x10^6^/ml for 2 hours. Cells were then fixed to the slide for 10 minutes with 4% formaldehyde in PBS-T (1x PBS with 0.01% Triton X-100) at room temperature and stored in PBS at 4°C until use. Cells were permeabilized in 0.5% Triton-PBS for 15 minutes. Cells were then dehydrated with an ethanol row, consisting of 2 minutes each in 70%, 90%, and 100% ethanol. Slides were dried for 1 minute, incubated for 5 minutes in 2x SSCT (0.3M NaCl, 0.03 M sodium citrate, and 0.1% Tween 20), and then incubated for 5 minutes in 2xSSCT/50% formamide at room temperature. The cells were then pre-denatured in 2xSSCT/50% formamide at 92°C for 2.5 minutes, then in 2xSSCT/50% formamide at 60°C for 20 minutes. Slides cooled for 5 minutes at room temperature. Primary Oligopaint probes in hybridization buffer (10% dextran sulfate, 2xSSCT, 50% formamide, 4% PVSA), 5.6mM dNTPs and 10µg RNaseA were then added to the slides, covered with a coverslip, and sealed with rubber cement. Slides were denatured at 92°C for 2.5 minutes once rubber cement was dry and then were transferred to a humidified chamber and incubated overnight at 37°C.

The following day, the coverslips were removed, and slides were washed in 2xSSCT at 60°C for 15 minutes, 2xSSCT at room temperature for 10 minutes, and 2xSSCT at room temperature for 10 minutes again. Next, hybridization buffer (10% dextran sulfate, 2xSSCT, 50% formamide, 4% PVSA) containing secondary probes conjugated to fluorophores was added to slides, covered with a coverslip, and sealed with rubber cement. Slides were incubated for two hours in a humidified chamber at room temperature protected from light. Slides were then incubated in 2xSSCT at 60°C for 15 minutes, and nutated in 2xSSCT at room temperature for 10 minutes twice. Slides were incubated with Hoescht (1:10,000 in 2xSSC) for 5 mins to stain DNA. Slides were then mounted using SlowFade Gold Antifade (Invitrogen).

### RNA-sequencing

10 million BG3 cells were harvested after dsRNA knockdown (described above). Cells were washed with 1x PBS and centrifuged at 600g for 5 minutes. Supernatant was aspirated off and the cell pellets were flash frozen and stored at -80°C. Cell pellets were shipped on dry ice to MedGenome. MedGenome extracted RNA and prepared libraries with Illumina Tru-Seq mRNA stranded kit. Libraries were sequenced on a Nova-seq with 100-bp paired end reads to a depth of at least 20 million reads.

To quantify expression of both genes and TEs, RNA-seq reads were adapter-trimmed using fastp (version 0.23.2) [41] and aligned to a reference genome consisting of consensus TE sequences appended to the repeat-masked BG3 assembly. Alignment was performed using hisat2 (version 2.2.1) [42] and raw read counts for genes and TEs were generated using htseq-count (version 0.11.2) [43].

Differential expression was assessed using DESeq2 (version 1.38.0) [44]. Shrunken log fold changes were generated using the DESeq2 lfcShrink function and the ashr estimator [45]. Normalized expression values were generated using the DESeq2 rlog function.

### Hi-C library preparation

Hi-C libraries were generated from 1x10^6^ BG3 cells using the Arima-HiC + kit (Arima Genomics) and the Kapa Hyper Prep Kit with the KAPA Library Amplification Primer Mix (KK8502) according to manufacturer’s recommendations. Sonication was performed on a Covaris M220 in 130µl microTUBE (Covaris), with the following program: Duration = 70s, Peak Power (W) = 50, Duty Factor (%) = 10, Cycles per Burst = 200. Libraries were validated for quality and size distribution using the Qubit dsDNA HS Assay Kit (Invitrogen, Q32851), the KAPA Library Quantification kit (Roche, KK4824) and the TapeStation 2200 (Agilent). Hi-C libraries were 100 bp paired-end sequenced on the NovaSeq 6000 system (Illumina) to a depth of 100–300 million reads by MedGenome.

### Hi-C data analysis

The two replicates of each condition were found to be highly concordant with HiCRep (S6 Table). For each chromosome, we calculated the correlation between replicates 1 and 2 using the parameters h = 20 and max distance = 20Mb. Average correlations per condition: Brown = 0.979, Cap-H2 = 0.970, SLMB = 0.988. Each sample in replicate 1 more closely correlated to the corresponding sample than the other samples in replicate 2. Therefore, the two replicates were combined for further analysis.

Hi-C reads were processed using HiC-Pro (v.2.11.1) [46] to obtain putative interactions with the default parameters, except for LIGATION_SITE = GATCGATC,GANTGATC,GANTANTC,GATCANTC and GENOME_FRAGMENT generated by digest_genome.py using “-r ^GATC G^ANTC” parameters. We recovered the following number of contacts for each condition: Brown (153,671,124), Cap-H2 (139,527,458), SLMB (135,663,084). For downstream analyses, the HiC-Pro output was converted to.hic files using the hicpro2juicebox.sh utility. These files were further converted to.cool files using “hic2cool” tool (https://github.com/4dn-dcic/hic2cool). Juicer dump was used to extract matrices at 5kb or 50kb resolution and at various normalizations for many of the following analyses [47].

#### Compartment calling.

For compartment analysis, HiC-Pro output was first converted to tag directories using the HOMER “makeTagDirectory” command. Then the eigenvector PC1 values were calculated using “runHiCpca.pl” command at 5 kb and 50 kb resolution. For 5 kb resolution, these parameters were used: “-res 5000 -superRes 10000 -cpu 8 -genome dm6 -min 0.4.” For 50kb resolution: “-res 50000 -superRes 100000 -cpu 8 -genome dm6 -min 0.05.” For the X chromosome, the analysis was done on two different halves: chrX:1–12250000 and chrX:12250001–23542271 because the initial settings were unable to detect the compartments. Contiguous bins of at least 20kb in size with PC1 values of the same sign were called A compartments if positive or B compartments if negative.

#### Trans-chromosomal interaction strength.

Using non-normalized Hi-C matrices at 500kb resolution, the total signal (sum) was calculated for each pair of chromosomes. The total for each pair of chromosomes was calculated as the proportion from the total signal from one chromosome, i.e., (Total signal from chr2L to chr2R)/(Total signal from chr2L to all).

#### Short-to-Long Range contact ratio.

The HiCExplorer tool hicPlotSVL was used to calculate the short to long range ratios [48]. 1Mb was used as the distance cutoff to consider an interaction short-range.

#### P(s) curves.

The cooltools notebook “Contacts vs Distance” was used to calculate the P(s) curve [49]. The compartments used were the custom compartment calls described above.

#### TAD calling.

The HiCExplorer tool hicFindTADs was used to call TADs with FDR as the multiple testing correction [48]. TAD size and number were calculated from the resulting “domains” bed file. Differential TAD boundaries were identified using the resulting “boundaries” bed file. Boundaries that shifted 10kb or less were categorized as “Same”, boundaries that shifted by 15–70kb were categorized as “Shift”, TADs in the control condition that were split into two TADs in the KD conditions were categorized as “Split”, and TADs from the control condition that were merged into a single TAD in the KD were categorized as “Merge”.

#### Interaction strength by distance.

Using KR normalized Hi-C matrices at 5kb resolution, bins were annotated as active, polycomb, or null. These designations came from Sexton and were lifted over to dm6 using UCSC Genome Browser “Lift Genome Annotations” tool (https://genome.ucsc.edu/cgi-bin/hgLiftOver). The BEDtools tool “intersect” was then used to annotate both coordinates of the Hi-C data with these chromatin type designations [50]. R was then used to filter the data by both chromatin type designations and distance to obtain the interactions strengths per chromatin type at 500kb and 1Mb.

#### Long-range compartment interactions.

The compartments called from brown (control) were numbered consecutively along the genome. Using KR normalized Hi-C matrices at 5kb resolution, bins were annotated based on their compartment number and compartment type in the brown (control) condition using BEDtools [50]. R was then used to group the data by compartment pair (i.e., all bins that represent interactions between compartment A1 and compartment A6). The mean of each compartment pair was calculated. These scores for Cap-H2 KD and SLMB KD conditions were then calculated as the fold-change to the Brown KD condition.

#### Virtual 4-C analyses.

Using KR-normalized, 5kb resolution data, anchors were manually defined and grouped as one large compartment (as in the long-range compartment interactions section above). The interaction score from the defined compartment was then calculated to each 5kb bin along the length of the same chromosome arm or to the opposite chromosome arm.

#### BG3-specific genome build.

High molecular weight (HMW) DNA was extracted from ~2.5 million BG3 cells using the NEB Monarch HMW genomic DNA extraction kit for Cells and Blood. The Oxford Nanopore Technologies (ONT) library prep kit SQK-LSK114 was used to prepare a sequencing library from the extracted DNA, which was run on a single R10.1.4 flow cell using the MinION sequencing instrument. Basecalling was performed using Guppy version 6.4.8 with the following model: dna_r10.4.1_e8.2_260bps_hac@v3.5.2.

All reads passing default Guppy QC were used as input for the Flye assembler (version 2.9.2) [51] which was run with the *--nano-hq* and *--no-alt-contigs* arguments and an estimated genome size of 205 Mb, to account for the Y chromosome. Flye contigs were scaffolded using the 3D-DNA pipeline (version 180922) [52] and the Hi-C data from control BG3 cells (described above). The *D. melanogaster* FlyBase gene annotations [53] were transferred to the new assembly coordinates using LiftOff (version 1.6.3) [54]. The BG3 assembly was compared to the dm6 reference genome assembly using the dnadiff utility from MUMmer 4.0 [55].

## Supporting information

S1 Fig Quantification and location of Cap-H2 levels and chromosome territory measurements. A. First panel: qPCR for Cap-H2 following Cap-H2 RNAi treatment. Four replicates yield an average KD to ~50% of endogenous levels. ∆∆CT was calculated to three housekeeping genes and normalized to control (brown KD). Second panel: qPCR for SLMB following SLMB RNAi treatment. Five replicates yield an average KD to ~17% of endogenous levels. ∆∆CT was calculated to three housekeeping genes and normalized to control (brown KD). B. First panel: Western blot to GFP-tagged Cap-H2 following transfection of Cap-H2:EGFP and RNAi. GFP expression was normalized to alpha tubulin control. Second panel: Quantification of western blot. Cap-H2 KD reduced Cap-H2:EGFP levels by 74% and SLMB increased Cap-H2:EGFP levels to 183%. C. Cumulative frequency histograms of chromosome 2L and chromosome 2R surface area for control (brown KD), Cap-H2 KD, and SLMB KD conditions. N: Control = 1229, Cap-H2 = 1,050, SLMB = 879. **** represents p < 0.0001 in Mann-Whitney test. D. ChIP-seq peak locations in 9 state chromatin model. Active chromatin types labeled in blue represent 44% (1119 peaks), repressed states in red represent 15% (376), and null in black represents 41% (1024).
(TIF)

S2 Fig Additional chromatin state FISH measurements. A. Cumulative frequency histograms representing the surface area of the segmented structures per nucleus for replicate 2. N: Control = 1185, Cap-H2 = 1601, SLMB = 979. **** represents p < 0.0001 in Mann-Whitney test. B. Number of individually segmented domains per nucleus. Bars represent the mean and error bars represent one standard deviation. For replicate 1, N: Control = 1419, Cap-H2 = 1331, SLMB = 743. **** represents p < 0.0001 in Mann-Whitney test. For replicate 2, N: Control = 1185, Cap-H2 = 1601, SLMB = 979. C. The reversed measurement from Fig 1G was calculated as the fraction of repressed volume overlapping with the active domain (first panel). N: Control = 1419, Cap-H2 = 1331, SLMB = 743. These measurements were also performed on replicate 2 (second and third panels). N: Control = 1185, Cap-H2 = 1601, SLMB = 979.
(TIF)

S3 Fig Hi-C interactions by chromatin state. A. Percent of total reads/condition reflecting interactions between each chromosome pair. B. Interaction strength between bins located 500kb apart where both bins are designated as active, repressed, or null. Hi-C data is at 5kb resolution and KR-normalized. **** represents p < 0.0001 in Mann-Whitney test as compared to control. *** represents p < 0.001 in Mann-Whitney test as compared to control. C. Interaction strength between bins located 5Mb apart where both bins are designated as active, repressed, or null. Hi-C data is at 5kb resolution and KR-normalized. **** represents p < 0.0001 in Mann-Whitney test as compared to control. ** represents p < 0.01 in Mann-Whitney test as compared to control.
(TIF)

S4 Fig Pairwise FISH by chromatin type. **A.** Schematic of 22 pairs of probed regions along chromosome 3R. Percent of cells with contacting probes (overlapping by at least one pixel) for each of 22 pairs of probed regions, ordered by genomic distance. N > 300 nuclei per condition. **B.** Minimum distance between probes for each of 22 pairs of probed regions, ordered by genomic distance. Median of >300 nuclei shown per condition. **C.** Map of two example pairs of probed regions located 0.2Mb and 5.4Mb apart. Mean and standard deviation of the percentage of cells in contact across two to four technical replicates. N > 300 nuclei per replicate. Example nuclei showing contact and no contact.
(TIF)

S5 Fig Pericentric genes expression in SLMB KD. A. Map of chromosome 3L PC1 eigenvector values for control, Cap-H2 KD, and SLMB KD. Below, zoom into the highlighted grey region within the pericentric domain. The gene vtd (highlighted in yellow) is located in a region that changes from negative eigenvector values in control to positive values in SLMB KD. B. Correlation of gene expression in control and Cap-H2 KD RNA-seq data. Only 4 genes were downregulated and 24 genes and 3 transposable elements were upregulated out of 8,806 detected features. C. qPCR results for three housekeeping genes and four compartment switch genes in the pericentric compartments. ∆∆CT is calculated to the geometric mean of the three housekeeping genes.
(TIF)

S6 Fig Definition of the pericentric heterochromatin domain. A. Scree plot for the k-means clustering of the eigenvectors. B. K-means clustering of the eigenvectors into three clusters. C. PC1 of the compartment eigenvector along the length of chromosome 2R for control (Brown KD, black), Cap-H2 KD (pink), and SLMB KD (blue). Highlighted yellow box represents the pericentric compartment. Positive values reflect A-compartment identity and negative values reflect B-compartment identity. D. Zoom in of the PC1 eigenvector across the pericentric domain (top 3 tracks). A compartment calls are shown on the bottom 3 tracks. E. Percent change of the average interaction score within the pericentric compartment and sized-matched controls along the chromosome arm.
(TIF)

S1 Table Chromosome state FISH probe information. A list of start and end genomic coordinates based on Dm3 genome build for each Oligopaint probe that was used to label different chromatin states (Active, Pc, Null) on Chr3R.
(XLSX)

S2 Table Pericentric heterochromatin regions. Genomic coordinates of the pericentric heterochromatin regions based Dm3 genome build for each Drosophial chromosome arm.
(XLSX)

S3 Table 100kb region FISH Probe information. A list of start and end genomic coordinates based on Dm3 genome build for each additional Oligopaint probe that was used to in this study.
(XLSX)

S4 Table Primer sequences used for dsRNA production. A list of forward and reverse primer sequences used for production of dsRNA for Brown (control), Cap-H2, and SLMB RNAi experiments.
(XLSX)

S5 Table Primer sequences used for qPCR. Forward and reverse primer sequences used for qPCR of Aldh7A1, P5CS, Ssadh, Cap-H2 (1 and 2), and SLMB.
(XLSX)

S6 Table Information related to Hi-C quality control metrics. Total read count pre- and post-processing for each step of Hi-C analysis for each condition and replicate.
(XLSX)
