# Supplementary figures and images for: The length and strength of compartmental interactions are modulated by condensin II activity
Source: PLoS Genet. 2025 Jul 1;21(7):e1011724. doi: 10.1371/journal.pgen.1011724 (PMC12244531; doi:10.1371/journal.pgen.1011724)

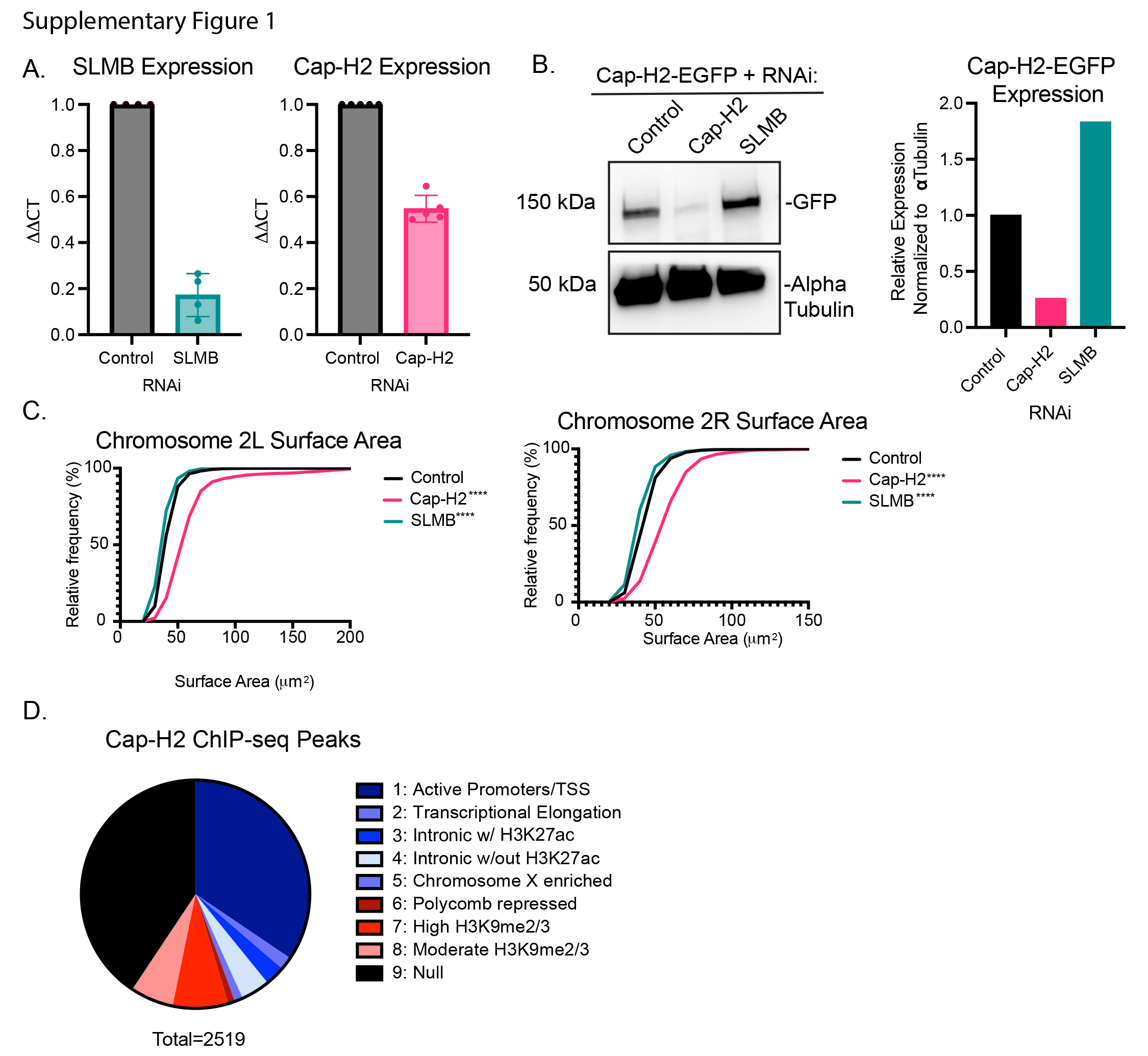

Supplement: S1 Fig — A. First panel: qPCR for Cap-H2 following Cap-H2 RNAi treatment. Four replicates yield an average KD to ~50% of endogenous levels. ∆∆CT was calculated to three housekeeping genes and normalized to control (brown KD). Second panel: qPCR for SLMB following SLMB RNAi treatment. Five replicates yield an average KD to ~17% of endogenous levels. ∆∆CT was calculated to three housekeeping genes and normalized to control (brown KD). B. First panel: Western blot to GFP-tagged Cap-H2 following transfection of Cap-H2:EGFP and RNAi. GFP expression was normalized to alpha tubulin control. Second panel: Quantification of western blot. Cap-H2 KD reduced Cap-H2:EGFP levels by 74% and SLMB increased Cap-H2:EGFP levels to 183%. C. Cumulative frequency histograms of chromosome 2L and chromosome 2R surface area for control (brown KD), Cap-H2 KD, and SLMB KD conditions. N: Control = 1229, Cap-H2 = 1,050, SLMB = 879. **** represents p < 0.0001 in Mann-Whitney test. D. ChIP-seq peak locations in 9 state chromatin model. Active chromatin types labeled in blue represent 44% (1119 peaks), repressed states in red represent 15% (376), and null in black represents 41% (1024). (TIF) [file pgen.1011724.s001.tif]

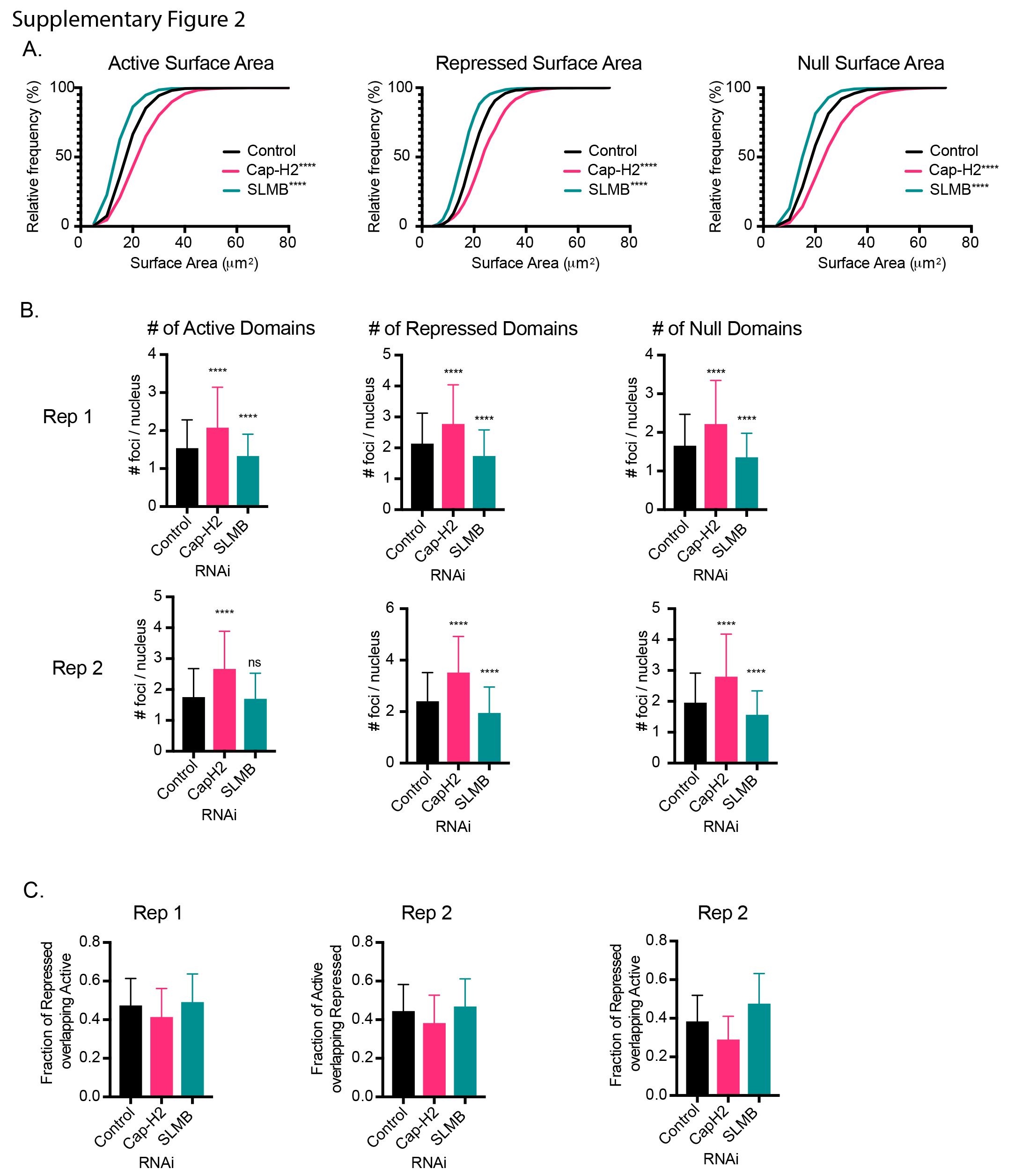

Supplement: S2 Fig — A. Cumulative frequency histograms representing the surface area of the segmented structures per nucleus for replicate 2. N: Control = 1185, Cap-H2 = 1601, SLMB = 979. **** represents p < 0.0001 in Mann-Whitney test. B. Number of individually segmented domains per nucleus. Bars represent the mean and error bars represent one standard deviation. For replicate 1, N: Control = 1419, Cap-H2 = 1331, SLMB = 743. **** represents p < 0.0001 in Mann-Whitney test. For replicate 2, N: Control = 1185, Cap-H2 = 1601, SLMB = 979. C. The reversed measurement from Fig 1G was calculated as the fraction of repressed volume overlapping with the active domain (first panel). N: Control = 1419, Cap-H2 = 1331, SLMB = 743. These measurements were also performed on replicate 2 (second and third panels). N: Control = 1185, Cap-H2 = 1601, SLMB = 979. (TIF) [file pgen.1011724.s002.tif]

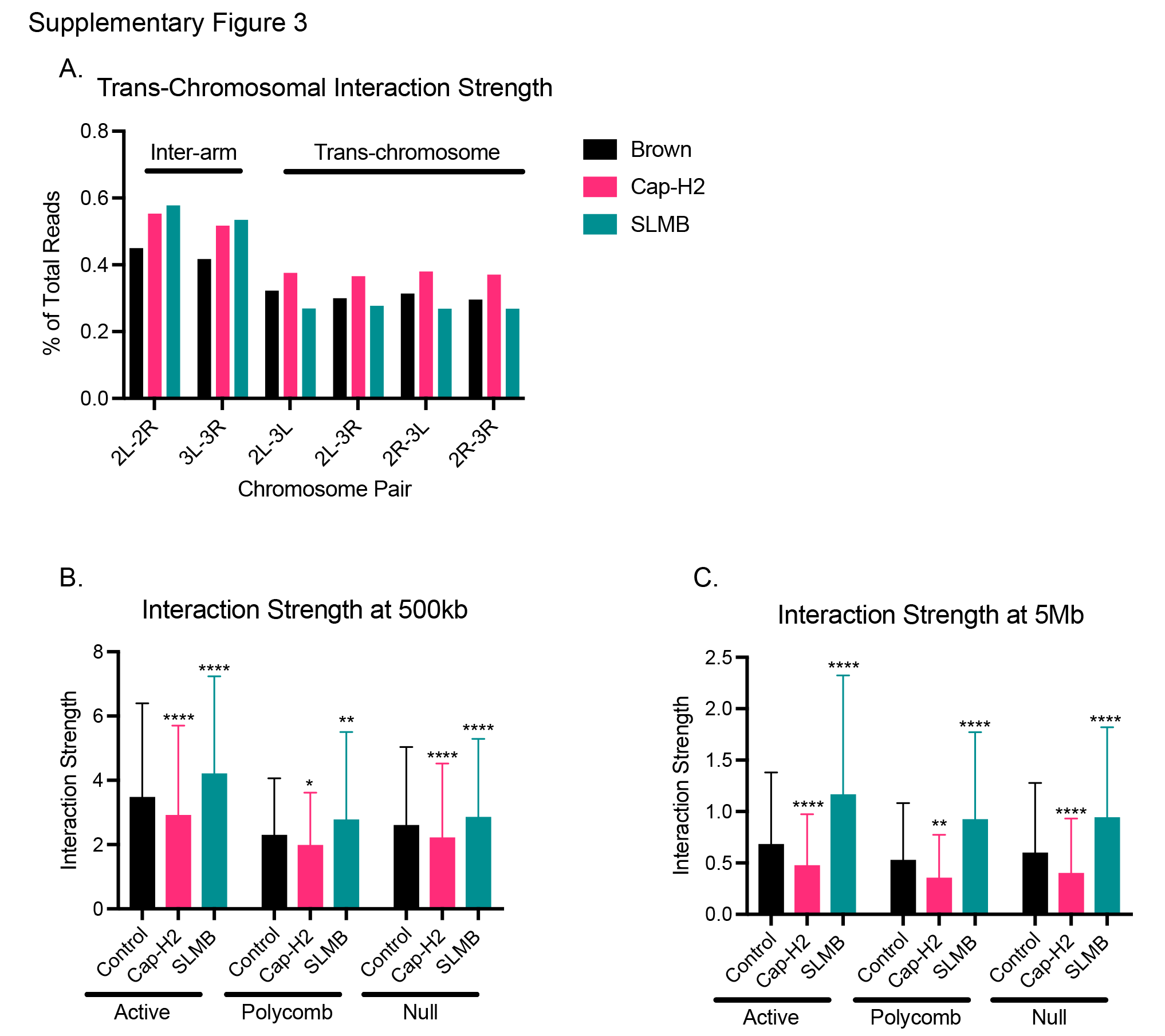

Supplement: S3 Fig — A. Percent of total reads/condition reflecting interactions between each chromosome pair. B. Interaction strength between bins located 500kb apart where both bins are designated as active, repressed, or null. Hi-C data is at 5kb resolution and KR-normalized. **** represents p < 0.0001 in Mann-Whitney test as compared to control. *** represents p < 0.001 in Mann-Whitney test as compared to control. C. Interaction strength between bins located 5Mb apart where both bins are designated as active, repressed, or null. Hi-C data is at 5kb resolution and KR-normalized. **** represents p < 0.0001 in Mann-Whitney test as compared to control. ** represents p < 0.01 in Mann-Whitney test as compared to control. (TIF) [file pgen.1011724.s003.tif]

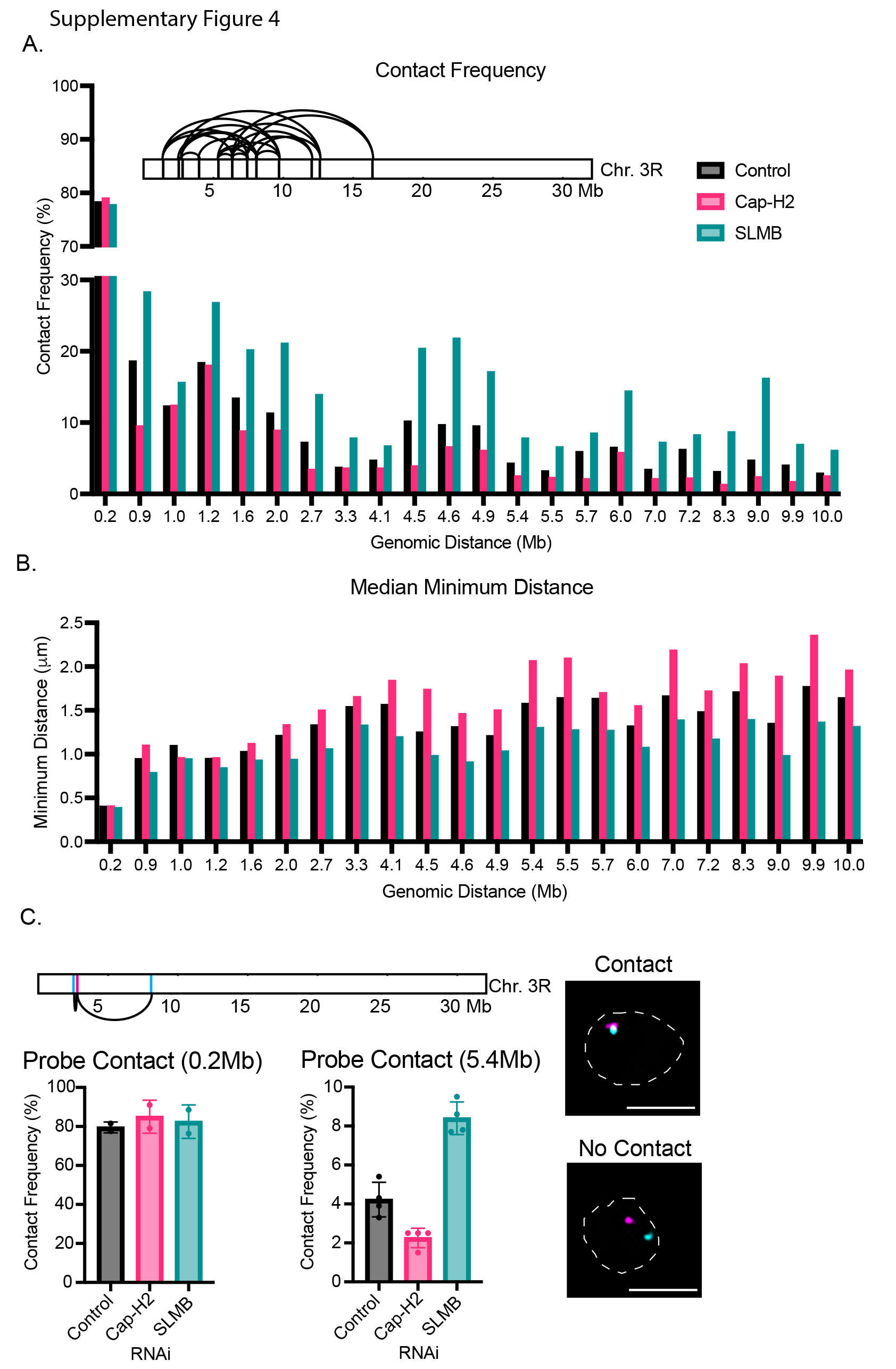

Supplement: S4 Fig — A. Schematic of 22 pairs of probed regions along chromosome 3R. Percent of cells with contacting probes (overlapping by at least one pixel) for each of 22 pairs of probed regions, ordered by genomic distance. N > 300 nuclei per condition. B. Minimum distance between probes for each of 22 pairs of probed regions, ordered by genomic distance. Median of >300 nuclei shown per condition. C. Map of two example pairs of probed regions located 0.2Mb and 5.4Mb apart. Mean and standard deviation of the percentage of cells in contact across two to four technical replicates. N > 300 nuclei per replicate. Example nuclei showing contact and no contact. (TIF) [file pgen.1011724.s004.tif]

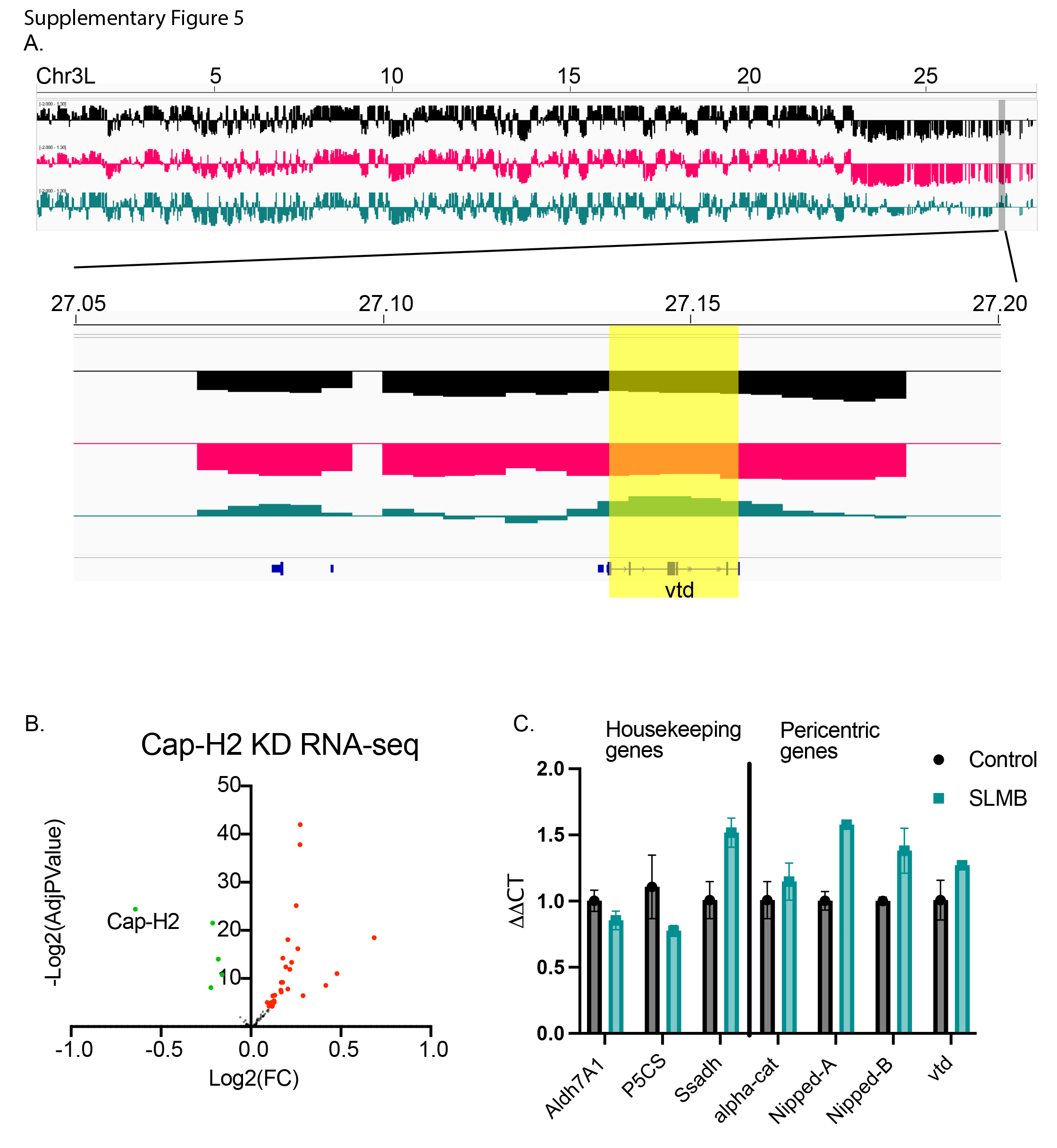

Supplement: S5 Fig — A. Map of chromosome 3L PC1 eigenvector values for control, Cap-H2 KD, and SLMB KD. Below, zoom into the highlighted grey region within the pericentric domain. The gene vtd (highlighted in yellow) is located in a region that changes from negative eigenvector values in control to positive values in SLMB KD. B. Correlation of gene expression in control and Cap-H2 KD RNA-seq data. Only 4 genes were downregulated and 24 genes and 3 transposable elements were upregulated out of 8,806 detected features. C. qPCR results for three housekeeping genes and four compartment switch genes in the pericentric compartments. ∆∆CT is calculated to the geometric mean of the three housekeeping genes. (TIF) [file pgen.1011724.s005.tif]

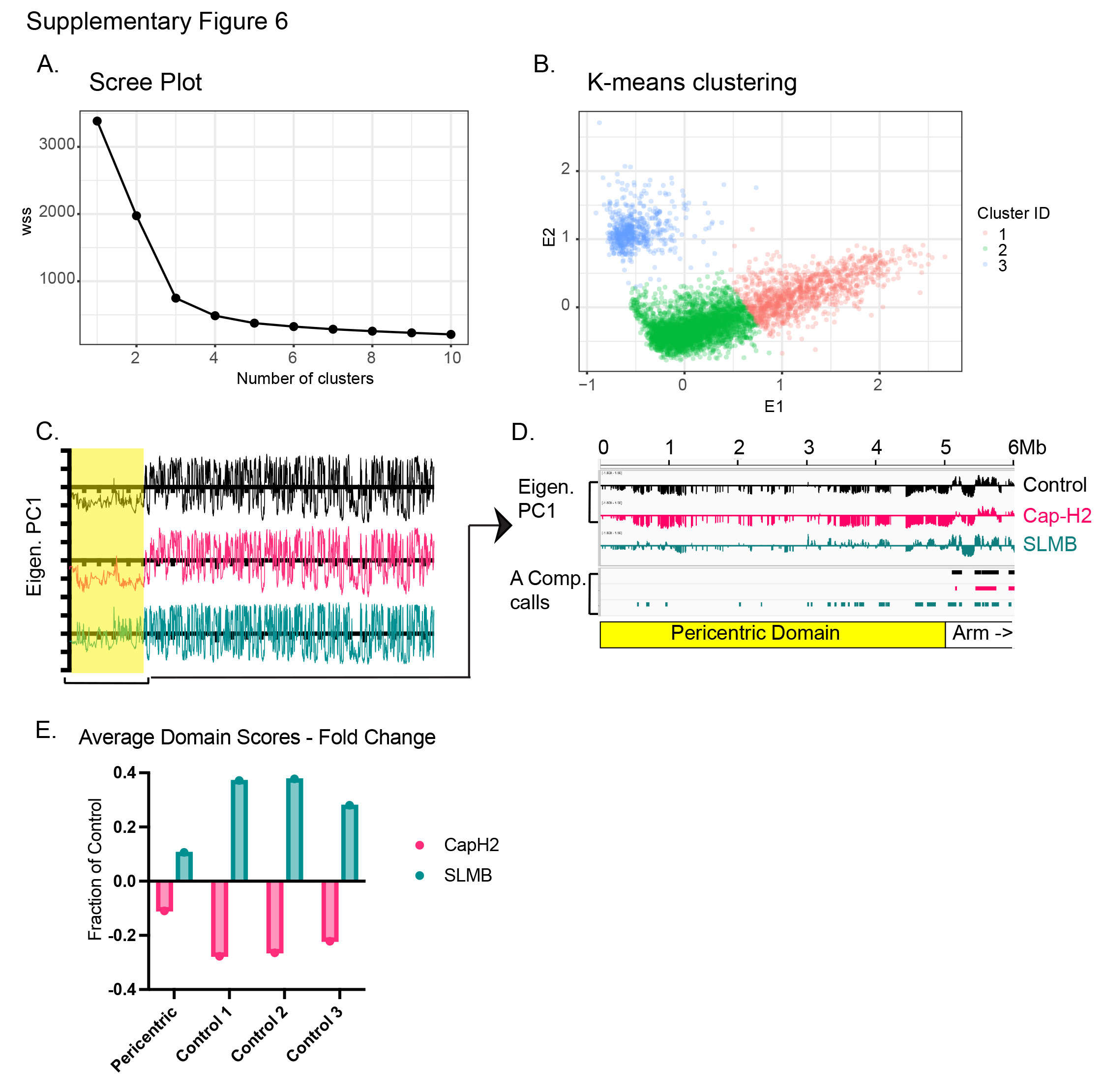

Supplement: S6 Fig — A. Scree plot for the k-means clustering of the eigenvectors. B. K-means clustering of the eigenvectors into three clusters. C. PC1 of the compartment eigenvector along the length of chromosome 2R for control (Brown KD, black), Cap-H2 KD (pink), and SLMB KD (blue). Highlighted yellow box represents the pericentric compartment. Positive values reflect A-compartment identity and negative values reflect B-compartment identity. D. Zoom in of the PC1 eigenvector across the pericentric domain (top 3 tracks). A compartment calls are shown on the bottom 3 tracks. E. Percent change of the average interaction score within the pericentric compartment and sized-matched controls along the chromosome arm. (TIF) [file pgen.1011724.s006.tif]
